# A Machine Learning Prediction Model to Identify Individuals at Risk of 5-Year Incident Stroke Based on Retinal Imaging

**DOI:** 10.3390/s25061917

**Published:** 2025-03-19

**Authors:** Arun Govindaiah, Tasin Bhuiyan, R. Theodore Smith, Mandip S. Dhamoon, Alauddin Bhuiyan

**Affiliations:** 1 iHealthScreen Inc., Richmond Hill, NY 11418, USA; arun@ihealthscreen.org (A.G.); tbhuiyan02@gmail.com (T.B.); 2 Biomolecular Retinal Imaging, Department of Ophthalmology, Icahn School of Medicine at Mount Sinai, New York, NY 10029, USA; rts1md@gmail.com; 3 Department of Neurology, Icahn School of Medicine at Mount Sinai, New York, NY 10029, USA; mandip.dhamoon@mssm.edu

**Keywords:** stroke, risk score, machine learning, AI in medicine

## Abstract

Stroke is a leading cause of death and disability in developed countries. We validated an AI-based prediction model for incident stroke using sensors such as fundus cameras and ophthalmoscopes for retinal images, along with socio-demographic data and traditional risk factors. The model was trained on a proprietary dataset of over 6500 participants, including 171 with 5-year incident strokes and 242 with 10-year incident strokes. The model provides separate 5-year and 10-year risk scores. The model was externally validated on the UK Biobank dataset (3000 subjects with 5-year incident strokes). Using retinal imaging, our models identified individuals with 5-year incident strokes with 80% sensitivity, 82% specificity, and an AUC of 0.83, and predicted 10-year incidents with 72% sensitivity, 78% specificity, and an AUC of 0.79. In comparison, for the 10-year model, the AUC for the Framingham score was 0.73, and the CHADS2 score was 0.74. On the Biobank external dataset, our 5-year model (without retinal features) demonstrated moderate but lower sensitivity (69.3%) and specificity (66.4%) compared to its performance on the proprietary dataset (with retinal features). Using a multi-ethnic dataset, we developed and validated a prediction model that improves stroke risk identification for 5-year and 10-year incidences by incorporating retinal features.

## 1. Introduction

Stroke, or cerebrovascular accident (CVA), kills almost 130,000 Americans each year, which is one out of every twenty deaths. On average, an American dies of a stroke every four minutes [1]. About 800,000 people in the United States have a stroke each year. Stroke costs the United States an estimated 34 billion USD each year. The incidence of this disease is expected to increase (due to an aging population and a rise in obesity), while the CDC reported that nearly one-fourth of the deaths due to strokes are avoidable [1,2]. Early identification of people at risk of stroke would allow earlier implementation of preventative strategies like medication and lifestyle changes to save lives, prevent disability, and save hundreds of thousands of dollars from each incident stroke [1]. The 34 billion USD per year total direct cost related to stroke could be lowered by 25% with appropriate protocols for stroke prevention [1,2].

To our knowledge, there is no effective screening tool or a stroke risk algorithm for predicting individuals at risk of stroke which is developed based on a diverse dataset. Our literature review found a number of stroke studies on specific population groups [3,4,5,6,7,8]. These models, however, did not predict an individual’s 5-year or 10-year incident risk, nor were the sensitivity and specificity of the models provided. We also observed that the published risk prediction models do not adequately capture a substantial proportion of people at high risk of stroke (e.g., the Framingham score [3] predicted only 47% of the morbidity [9], lacked precision in risk estimation, and has other limitations [10]). There is thus a need for greater prediction accuracy and a screening tool. Furthermore, the published stroke prediction models [9,10] lack innovative retinal image analysis algorithms, even though recent studies have shown that retinal vessel features (such as vessel width and focal arteriolar narrowing) are statistically significantly associated with stroke [11,12]. Motivated by this unmet need, our system included retinal imaging (color fundus imaging, the ophthalmoscope/fundus camera as the sensor) to improve patient awareness and potential clinical application.

## 2. Materials and Methods

### 2.1. Data Sources

**Figure A1:**
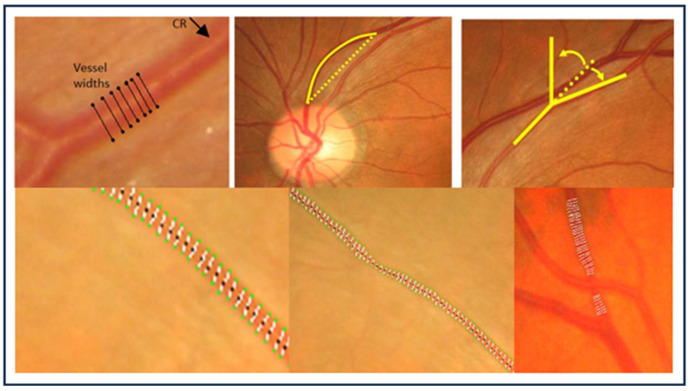
From top left: vessel widths and the central light reflex (black arrow); the vessel tortuosity (yellow curve); and the branch angle (yellow dashed lines) on full yellow lines showing the vessels. From bottom left: quantified vessel width, FAN, and AVN.

We used data from multi-ethnic participants from various data sources and constructed the dataset to build the stroke prediction model. We computed quantified retinal features (sample images are provided in Figure A1 in the Appendix A) such as vessel width [13], arteriovenous (AV) nicking [14], microaneurysms [15], and hemorrhages [15] along with socio-demographic features such as age, gender, race, education level, income level, and conventional risk factors such as BMI, waist-to-hip ratio (WHR), smoking history, and diabetes and hypertension status, both significantly associated with stroke [16]. The features were derived from the baseline data collected at the start of the study. Stroke vs. non-stroke classes were defined based on whether participants were diagnosed with a stroke within 5 years from the baseline (incident stroke) or remained stroke-free during this period.

The proprietary dataset has over 6500 subjects with 5-year and 10-year incident stroke. Table 1 shows the baseline characteristics of the study participants on the incident stroke. From the participants, we took the medical history, retinal data, and socio-demographic data for 70% of the people for training the model and 30% for testing the system. We obtained an IRB (from Western IRB, #1-998255-1) to perform this AI-based stroke prediction study.

For external validation of the stroke prediction model, the UK Biobank [17] collected extensive baseline questionnaire data, physical measurements, and biological samples from 500,000 men and women aged 40–69 at baseline between 2006 and 2010 in 22 centers across the UK. In the ongoing study, it re-contacted subjects for follow-up information. Out of this population-based dataset, we chose only subjects who had 10-year follow-up data.

The following subsections describe the study datasets and methodologies for the development of the stroke prediction model.

### 2.2. The 5-Year Stroke Prediction Model

The 5-year stroke prediction model combines socio-demographic, medical, and retinal features to assess an individual’s risk of stroke within five years. The model was trained on a proprietary dataset of 5363 participants, including 171 individuals who experienced a stroke within five years. It utilizes Cox proportional hazards regression to identify significant features and employs an ensemble of machine learning classifiers [18] for enhanced prediction accuracy. This is a technique we have used previously with good effect in survival analysis in Age-related Macular Degeneration (AMD) [19], so we chose this route again.

#### 2.2.1. Data Preparation

Participants: The proprietary dataset consisted of 5363 participants, with 121 stroke cases and 3673 non-stroke cases allocated to the training set. The test set included 50 stroke cases and 1489 non-stroke cases. Participants were selected based on the availability of complete data across all features.

Features: A total of 17 features were used, including the following:

- Socio-demographic: age, gender, race, education, and income levels.
- Medical: BMI, waist-to-hip ratio, hypertension status, diabetes, smoking history, and lipid profiles (e.g., HDL and LDL cholesterol).
- Retinal: quantitative features such as vessel width, arteriovenous nicking, microaneurysms, and retinal hemorrhages.

#### 2.2.2. Feature Selection Using Cox Regression

To address data imbalance and identify the most significant features, Cox proportional hazards [18] regression was applied. Features with a *p*-value < 0.05 were selected, ensuring that only meaningful features contributed to model development. The Cox regression parameters (beta coefficients) were used to compute a risk score for each participant, defined as the sum of the products of the beta coefficients and the corresponding feature values.

**Figure 1:**
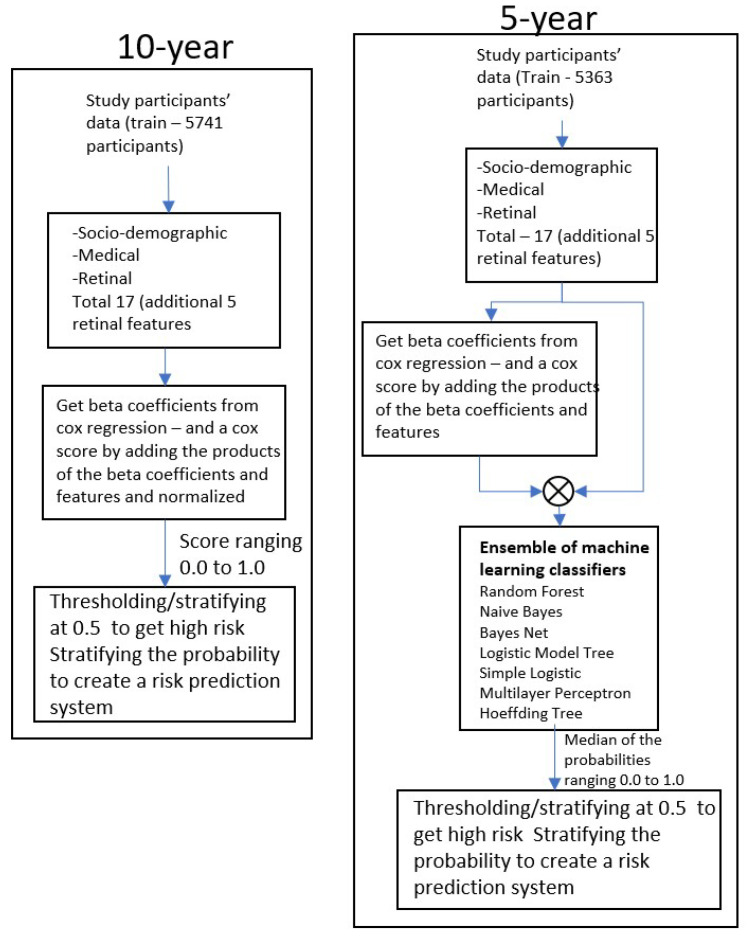
High-level view of the 5-year and 10-year stroke risk prediction methodology. Retinal and socio-demographic features along with medical history form the input which is then analyzed through the Cox hazards model and the coefficients are used to build machine learning models through different classifiers. The continuous variable is then stratified to create a risk prediction system through a binary classifier.

To enhance predictive accuracy and address the limitations of imbalanced data, the Cox risk scores, derived from significant features with *p*-values < 0.05, were utilized as inputs for an ensemble of machine learning classifiers. An ensemble of machine learning models often outperforms individual constituent models [20], so through experimentation, we selected seven best-performing models and ensembled them for 5-year stroke risk prediction. Seven classifiers were employed, including the Random forest [21], Naïve Bayes [22], BayesNet [23], Logistic Model Tree (LMT) [24], Simple Logistic [25], Multi-layer Perceptron [26], and Hoeffding Tree [27]. Each classifier was trained independently, leveraging the Cox-derived risk scores as a feature. To achieve a robust and reliable final prediction, the probabilities output by these classifiers were combined by calculating the median probability for each participant. This approach ensured that the final risk score, ranging from 0.0 to 1.0, captured the collective strengths of all classifiers, minimizing the risk of overfitting or bias from individual models. This score was thresholded to obtain the best sensitivity and specificity. The threshold was found to be 0.5. This ensemble method significantly improved the overall model performance and allowed for the stratification of participants into high-risk, medium-risk, and low-risk categories with improved sensitivity and specificity. A high level view of this process is shown in Figure 1.

Table A2 (in the Appendix A) shows the list of features selected by applying this procedure for each model.

### 2.3. The 10-Year Stroke Prediction Model

The 10-year stroke prediction model is designed to assess an individual’s risk of experiencing a stroke within a 10-year period. The model uses socio-demographic, medical, and retinal features to compute risk scores. Unlike the 5-year model, this system relies solely on Cox proportional hazards regression for both feature selection and risk score calculation, as additional machine learning approaches did not significantly improve the predictive performance.

#### 2.3.1. Data Preparation

Participants: The model was developed using a proprietary dataset of 5741 participants, of which 242 individuals experienced a stroke within 10 years. The dataset was split into training and testing sets, with 187 stroke cases and 3820 non-stroke cases used for training, and 55 stroke cases and 1679 non-stroke cases reserved for testing.

Features: A total of 17 features were included in the model, categorized as follows:

- Socio-demographic: age, gender, and race.
- Medical: BMI, hypertension status, diabetes, smoking history, and lipid profiles (HDL, LDL, and total cholesterol).
- Retinal: features such as vessel width, arteriovenous nicking, and retinal microvascular abnormalities.

#### 2.3.2. Feature Selection Using Cox Regression

To identify the most predictive features, Cox proportional hazards regression was employed. Features with *p*-values < 0.05 were retained for the final model. This regression method ensured that only statistically significant and clinically relevant features contributed to the risk assessment. The coefficients obtained from the Cox model were then used to calculate individual risk scores.

#### 2.3.3. Cox Risk Score Calculation

The risk score for each participant was computed as the sum of the products of the Cox regression coefficients and the corresponding feature values. These scores were normalized to a range of 0.0 to 1.0 for ease of interpretation and comparison across individuals. This score was thresholded to obtain the best sensitivity and specificity for positive and negative cases. The threshold was found to be 0.5.

## 3. Results

### 3.1. The 5-Year Incident Stroke Prediction Model Results

Table A3 (Appendix A) shows the number of subjects in training and testing sets for the 5-year prediction algorithm. From 5363 subjects, the number of 5-year incident stroke subjects with complete parameter data was 171. Out of these, we took 3673 non-stroke subjects and 121 stroke subjects for training the model, and 1419 non-stroke subjects and 50 stroke subjects for testing.

Table 2 shows a comparison of the 5-year stroke prediction models that did or did not include retinal features, and likewise for the 10-year models. Including retinal features improves the performance of the 5-year system, as evident from the increased sensitivity of 0.80 (CI—0.66 to 0.90) compared to 0.76 (CI 0.62 to 0.87).

### 3.2. The 10-Year Incident Stroke Prediction Model Results

Table A4 (Appendix A) shows the number of subjects in training and testing sets for the 10-year prediction algorithm. For the ten-year model development and validation, we have a total population of 5741 people. The number of stroke subjects is 242 with these features. Out of these, 187 subjects are taken for training the model and 55 for testing. Similarly, 3820 normal or non-stroke subjects were taken for training and 1679 for testing.

Like the 5-year model, the 10-year risk prediction system fares better, as evident from Table 2, when retinal features are included.

### 3.3. Comparison of the New iHS Model with Framingham Stroke and CHADS2 Scores (10-Year Models Such as Framingham and CHADS2 Provide 10-Year Risk Scores)

**Figure 2:**
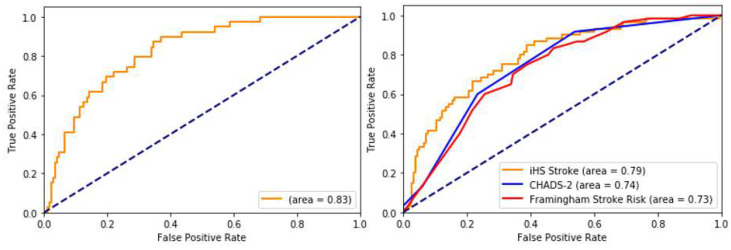
ROC for 5-year risk of stroke (**left**); comparison of ROCs for 10-year risk of stroke with CHADS2 and Framingham Stroke Risk Scores (**right**). Blue dashed line indicates line of no-discrimination or random classifier.

**Figure A2:**
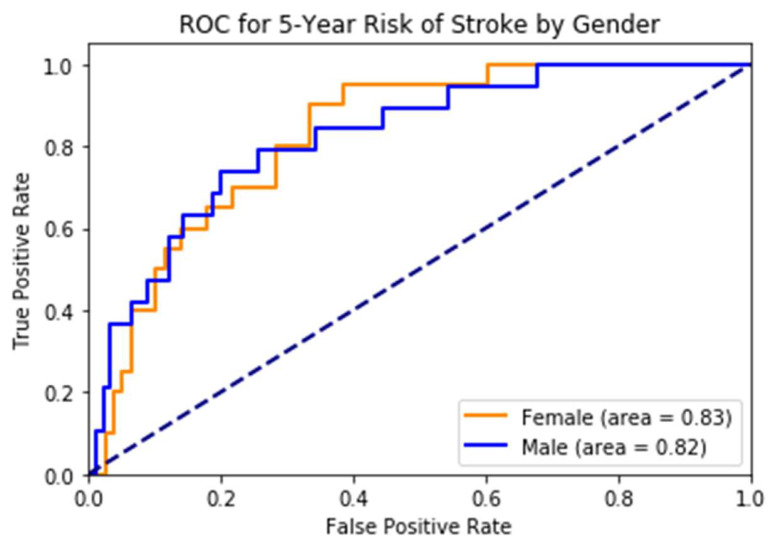
ROC for 5-Year Risk of Stroke stratified by Gender. Blue dashed line indicates line of no-discrimination or random classifier.

**Figure A3:**
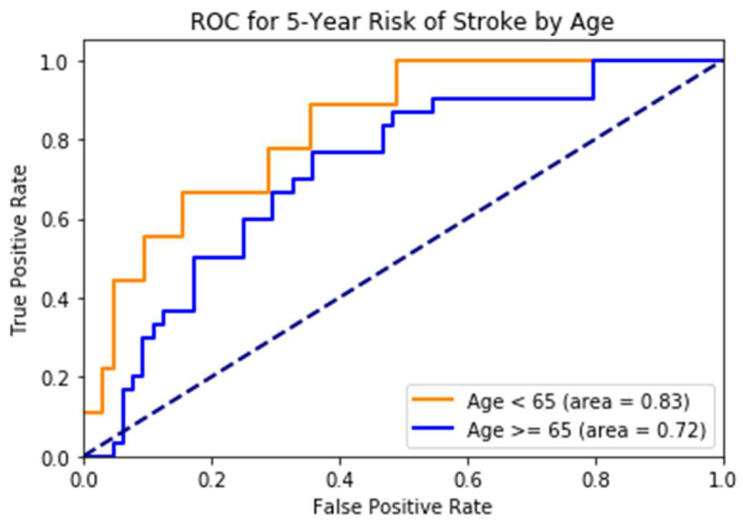
ROC for 5-Year Risk of Stroke stratified by Age. Blue dashed line indicates line of no-discrimination or random classifier.

**Figure A4:**
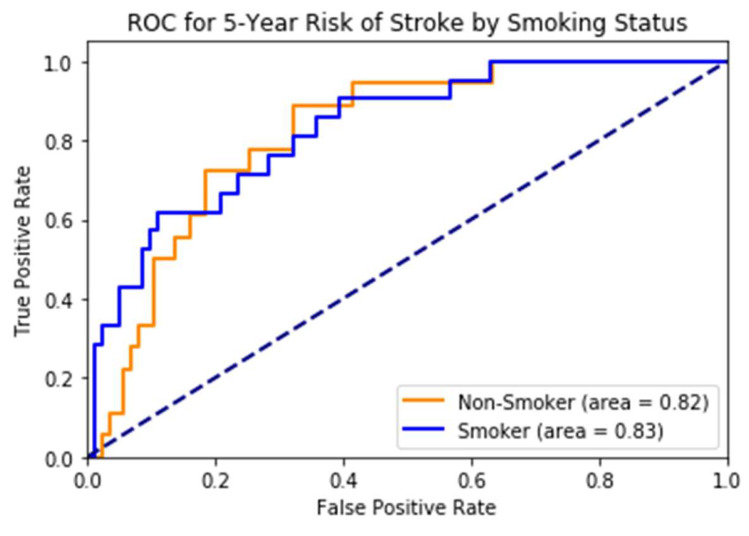
ROC for 5-Year Risk of Stroke stratified by Smoking Status. Blue dashed line indicates line of no-discrimination or random classifier.

**Figure A5:**
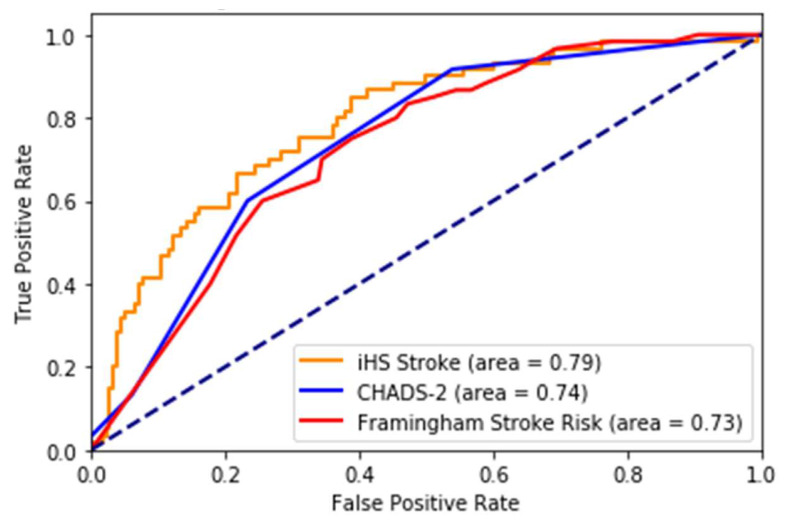
ROC for 10-Year Risk of Stroke ROC compared with CHADS-2 and Framingham Stroke Risk. Blue dashed line indicates line of no-discrimination or random classifier.

**Figure A6:**
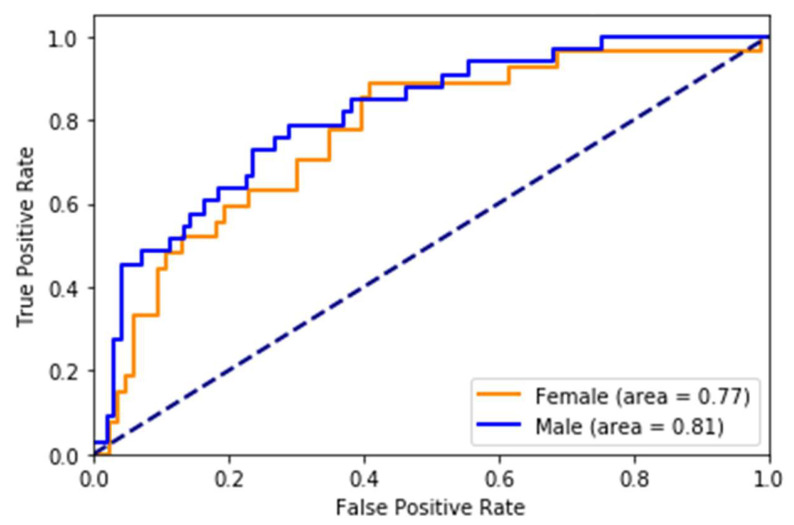
ROC for 10-Year Risk of Stroke stratified by Gender. Blue dashed line indicates line of no-discrimination or random classifier.

**Figure A7:**
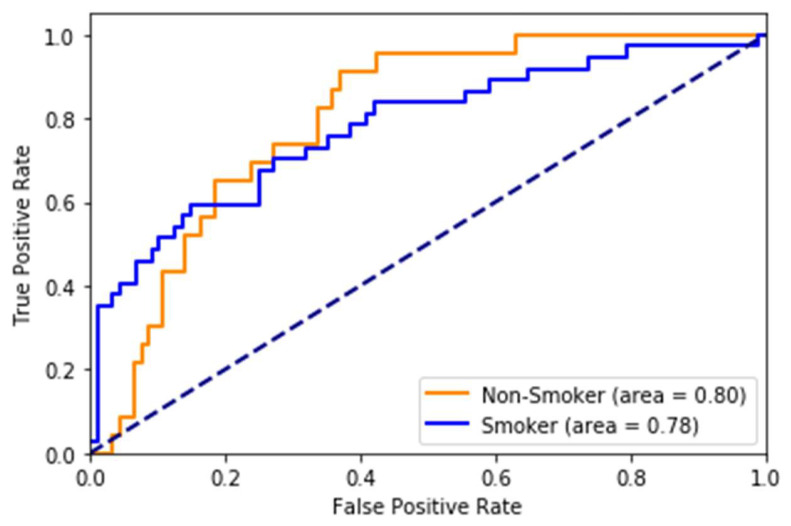
ROC for 10-Year Risk of Stroke stratified by Smoking Status. Blue dashed line indicates line of no-discrimination or random classifier.

**Figure A8:**
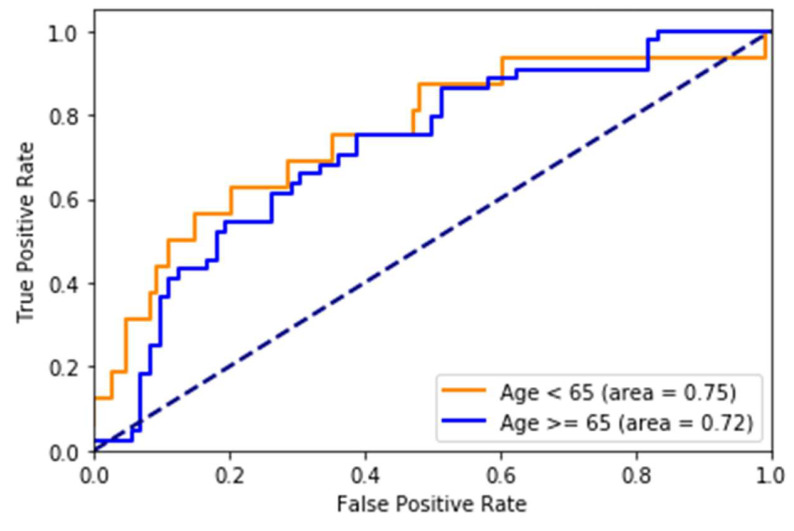
ROC for 10-Year Risk of Stroke stratified by Age. Blue dashed line indicates line of no-discrimination or random classifier.

We compared the prediction accuracy of our iHealthScreen (iHS) model with the well-known Framingham and CHADS2 [28] scores at 10 years. We acknowledge that we used the same test subjects for all three. Figure 2, Figure A2, Figure A3, Figure A4, Figure A5, Figure A6, Figure A7 and Figure A8 (Appendix A) and Table A5 (Appendix A) show the Receiver Operating Characteristic (ROC) curve comparisons of our prediction system with the Framingham and CHADS2 scores. Our proposed score outperformed the others with an AUC of 0.79 (0.78 to 0.80). We have also computed the risk score along with the sensitivity and specificity for each of these scores for the given test subjects. From Table 3, it is clear that at any cut-off risk, the iHS Score outperforms the other risk scores in terms of sensitivities and specificities. For example, consider the sensitivity of 80%: for the same sensitivity of 80%, the specificity of our model is 75%, and for Framingham, it is 61.7%. Similarly, for the sensitivity of 60% (in blue), the specificity of our model is 85%, and the specificity of CHADS2 is 76.7%.

The 5-year model without retinal parameter input has given promising results on the UK Biobank data as well. Our proposed model achieved a sensitivity of 69.3%) and a specificity of 66.4%. The significant improvement of the model on the proprietary dataset with retinal features, compared to without, however, suggests that the addition of retinal features would be helpful from other datasets where possible.

The 5-year and 10-year risk prediction models were also analyzed for their performance on race-stratified data. Our proposed 10-year incident stroke prediction model was also compared to the other two 10-year risk prediction models using the Framingham and CHADS2 Score. The 5-year incident stroke prediction model achieved an AUC of 0.77 in the white/Caucasian population. The AUC decreases to 0.68 for African Americans, prompting the importance of a race-adjusted separate model (Table 4).

Our 10-year model performs better than CHADS2 and Framingham. The AUC on the white/Caucasian data was 0.77 for our model compared to 0.68 for both the Framingham and CHADS2 models. For the Chinese Americans, all three models perform similarly to each other with the AUC of 0.64 to 0.66. However, all models fair worse for Hispanic and African American data. The AUC for the Framingham score is 0.4 for African Americans. The AUC for the CHADS2 model is 0.6 for Hispanics. We note that the Framingham and CHADS2 scores do not provide positive or negative cases. To compare with our model, we considered a cut-off of the top 20% for each score on the training data as positive cases (i.e., stroke positive cases) to measure the sensitivity and specificity. The full results of this exercise are shown in Table 5.

## 4. Discussion

The application of AI to clinical medicine is in its infancy, and several steps remain for meaningful clinical use. For stroke prevention, the first step is to determine whether an AI prediction model can be developed at all which is an improvement on known clinical models. This paper has demonstrated such a system with satisfactory performance on large, well-characterized existing datasets. At this point, however, one might want to focus on people who would not otherwise have an indication for stroke risk-reducing therapy based on current guidelines (e.g., hypertension, high ASCVD risk, atrial fibrillation). Thus, an immediate application could be an advisory tool to a patient with a high risk for stroke in 5 years, which the patient and physician could accept or reject, about the modifiable risk factors: diet, exercise, and BP control, with the understanding that definitive prospective proof of benefit is pending. The same philosophy has guided our creation of a prediction model for AMD based on the AREDS dataset, with the understanding that prospective validation is still required in studies that are now ongoing.

In this paper, we describe a novel and accurate artificial intelligence and retinal imaging-based stroke prediction model for screening an individual at risk of incident stroke, which offers significantly improved accuracy over traditional risk-prediction algorithms. The data from the study, which is an ongoing prospective observational study, were analyzed retrospectively to build the model. We achieved very good performance on 5-year and 10-year incident stroke prediction. While we acknowledge that the CHADS2 and Framingham scores were developed with different purposes and populations, they are the prevailing methods of stroke risk estimation and screening. We have shown that our method performs better than the Framingham score and CHADS2 score for stroke prediction, the best models among the reported stroke prediction scores.

While most stroke scores are based on the white population, we used multi-ethnic population-based data, which is one of the strengths of the study. The risk scores developed on (mostly) white cohorts will probably have limited generalizability to other races/ethnicities. Therefore, it is an excellent opportunity for us to take advantage of this multi-ethnic dataset. However, there is still a lot of room for improvement in this area. The race-wise results show the model still lacks calibration to all races/ethnicities. The risk prediction system, when used to predict stroke as a binary class (yes/no), may capture a high number of false positives or a high number of false negatives depending on the threshold value. We also note while the system with eye imaging features/measures performs better than the one without, these measures are not easily obtainable. They require expert photography and ophthalmologists to grade the retinal photos. This challenge can be an area for more research into the deep learning-based automated grading of retinal pathologies.

This study has several limitations. One of the main challenges was the imbalance of stroke and non-stroke subjects, with the relatively smaller number of stroke subjects posing a significant challenge for machine learning techniques. This limitation prompted us to use a combination of the Cox proportional hazards model and traditional machine learning classifiers. We took inspiration from Transfer learning, which is a technique popular in image classification problems where knowledge/features learned from large datasets are used in problems with similar but smaller datasets.

The Cox proportional hazards model was found to work better with unbalanced data than common machine learning classifiers on this data. We first extracted the significant features by applying the Cox method to the entire training data and then used them with machine learning classifiers with smaller balanced datasets. This procedure helped us keep the maximum number of sample types in each class at the beginning of training: all the 3794 subjects in the 5-year model and 4007 subjects in the 10-year model.

Another challenge was the missing features. Many data fields had an incorrect format unclear field values, or sometimes missing field values/data. The elimination of all records that have any missing or incorrect data would have resulted in significant data and information loss. Instead, we first extracted predictive features with all the features using a high threshold for *p*-value in the Cox proportional hazards model. Considering then only the selected features, we eliminated the records with missing or incorrect data. This allowed for a low data loss of about 15% (from 6814 records to 5741 records).

The validation dataset from UK Biobank is an extensive database covering diverse populations across the UK. The stroke prediction model proposed in this paper consistently performed on this dataset, making it viable for implementation. It also outperformed popular risk prediction scores such as the Framingham stroke risk score and CHADS-2 score for 5-year risk. With eye features, our prediction model showed an AUC of 0.83, compared to Framingham, 0.68, and CHADS2, 0.67, respectively. Because Framingham and CHADS2 do not use retinal features, this makes a good case for including them in stroke prediction models. Although our focus is mainly on 5-year stroke prediction, the results also showed that our model outperformed Framingham and CHADS2 for 10-year incident stroke risk.

Future work will focus on analyzing racial/ethnic differences in model performance, evaluating performance in specific high-risk subgroups, and incorporating additional calibration metrics. We will also explore strategies for periodic model recalibration to ensure consistent accuracy across diverse populations. Addressing these aspects will help improve the model’s generalizability and clinical utility.

## 5. Conclusions

We have used retinal blood vessels and pathological information, which provided us with higher sensitivity in 5-year and 10-year incident strokes. To the best of our knowledge, this is the best performance in stroke prediction. The proprietary dataset and UK Biobank datasets are retrospective datasets, and following a prospective trial, we aim to deploy the prediction model in the clinical settings for identifying stroke risk patients and helping take preventative measures more effectively. Another application of the model is to plug it into the telemedicine platform, which will help to overcome the computational power of the local clinics, which use tablets or small computers, and make it suitable for mass screening of the population to identify the risk of incident stroke.

## 6. Patents

Dr. Bhuiyan reports a patent on ‘Image-Based Screening System for Prediction of Individual at Risk of Stroke’ related to this paper.

## Figures and Tables

**Table 1:** Baseline characteristics of participants in the study according to their incident stroke status.

Characteristics (Means or Prevalences)	Stroke (N = 233)	Non-Stroke (N = 6478)
Age, years ± SD ^1^	68 ± 10	62 ± 10
Male, n (%)	111 (48)	3056 (47)
Non-Hispanic white American	85 (37)	2509 (39)
Chinese American	17 (7)	780 (12)
African American	66 (28)	1783 (28)
Hispanic American	65 (28)	1406 (22)
Controlled Hypertensive, SBP ^2^ < 140 and DBP ^3^ < 90 mmHg	119 (51)	2129 (33)
Uncontrolled Hypertensive, SBP ≥ 140 or DBP ≥ 90 mmHg	43 (19)	710 (11)
Diabetes, n (%)	57 (25)	776 (12)
Current smoker, n (%)	34 (15)	834 (13)
Body mass index, kg/m2 ± SD	29 ± 5	28 ± 6
Lipid-lowering medication use, n (%)	44 (19)	1039 (16)
Total cholesterol, mg/dL ± SD	196 ± 33	194 ± 36
High-density lipoprotein cholesterol, mg/dL ± SD	49 ± 14	51 ± 15
Low-density lipoprotein cholesterol, mg/dL ± SD	119 ± 29	117 ± 32
Triglycerides, mg/dL ± SD	140 ± 75	131 ± 90
SBP, mmHg ± SD	139 ± 23	126 ± 21
DBP, mmHg ± SD	74 ± 11	72 ± 10
Anti-hypertensive medication use, n (%) Hypertension categories, n (%)	126 (54)	2361 (37)
Normotensive, SBP < 140 and DBP < 90 mmHg	70 (30)	3639 (56)

Note: ^1^ SD indicates standard deviation; ^2^ SBP, systolic blood pressure; and ^3^ DBP, diastolic blood pressure.

**Table 2:** Comparison of sensitivity and specificity of the 5-year and 10-year stroke prediction models with and without considering eye features, showing 95% CI in braces.

	5-Year Stroke Prediction		10-Year Stroke Prediction	
Model	With Eye Features	Without Eye Features	With Eye Features	Without Eye Features
Sensitivity	0.80 (0.66 to 0.90)	0.76 (0.62 to 0.87)	0.73 (0.59 to 0.84)	0.72 (0.59 to 0.84)
Specificity	0.82 (0.69 to 0.91)	0.80 (0.66 to 0.90)	0.78 (0.65 to 0.88)	0.75 (0.61 to 0.85)

**Table 3:** iHS Stroke Risk Prediction Results on the proprietary dataset compared with CHADS2 and Framingham score. Scores stratified as high, medium, and low, along with the associated risk percentage, sensitivity, and specificity, are reported. Numbers in bold to show best model.

	Risk Category	Score	Risk Percentage (%)	Sensitivity (%)	Specificity (%)
iHS Stroke Score	High	0.9	15.9	41.7	91.7
		0.8	12.3	58.3	85.0
		0.7	13.0	68.3	83.3
		0.6	11.4	71.7	80.0
	Medium	0.5	10.2	**80.0**	**75.0**
		0.4	7.8	81.7	66.7
		0.3	6.9	90.0	58.3
	Low	0.2	6.3	93.3	53.3
		0	3.2	100.0	0.0
CHADS2 Score	High	6.0	18.2	1.7	100.0
		4.0	8.5	3.3	100.0
		3.0	5.9	13.3	91.7
	Medium	2.0	4.0	60.0	76.7
		1.0	2.8	91.7	50.0
	Low	0.0	1.9	100.0	0.0
Framingham Risk Score	High	>26	>30	51.7	83.3
		>21	>30	65.0	75.0
		>16	25 (men), 15 (women)	80.0	61.7
	Medium	>11	11 (men), 7 (women)	86.7	48.3
		>6	4.7 (men), 3.3 (women)	98.3	25.0
	Low	>0	1.4 (men), 1 (women)	100.0	0.0

**Table 4:** Analysis of 5-year stroke prediction models on the 5-year test data with measures of AUC, sensitivity, and specificity.

Race/Ethnicity	AUC	Sensitivity (%)	Specificity (%)
White	0.77	72.0	63.4
Chinese American	0.73	50.0	69.6
African Americans	0.68	64.5	56.7
Hispanic	0.70	70.5	57.5

**Table 5:** Analysis and comparison of 10-year stroke prediction models on the 10-year test data with the following measures: AUC (area under the curve), sensitivity, and specificity. Threshold points are not given for Framingham and CHADS2 scores. Therefore, a cut-off of the top 20% (in the training sets) is taken for measuring sensitivity and specificity.

Race/Ethnicity	Risk Scores	AUC	Sensitivity (%)	Specificity (%)
White/Caucasian	CHADS2	0.68	50.0	77.8
	Framingham	0.68	50.0	72.2
	iHS	0.77	57.1	80.6
Chinese American	CHADS2	0.66	58.3	80.6
	Framingham	0.64	53.8	78.1
	iHS	0.66	55.6	50.0
Black, African-American	CHADS2	0.52	38.1	55.2
	Framingham	0.40	42.9	51.7
	iHS	0.58	52.4	58.6
Hispanic	CHADS2	0.60	30.0	100.0
	Framingham	0.50	40.0	100.0
	iHS	0.50	40.0	100.0

## Data Availability

UK biobank is a publicly available dataset and can be obtained by applying at https://biobank.ndph.ox.ac.uk/showcase/ (accessed on 1 October 2024).

## Appendix A

**Table A1:** Categories of features in the dataset.

A variety of patient information during the study through questionnaires and assessments/procedures, including the following: Blood pressure, Electrocardiography, Anthropometry, Urine sample collection, Phlebotomy, Snack, Carotid ultrasound, Ankle–brachial index, Endothelial function, Arterial waveform collection, Medical history, Personal history, Demographics, Socio-economic status, Medications, Psycho-social assessment, Diet assessment, Physical activity, Family history, Clinic Exit/Tracking Retinal photographs of both eyes of the participants were obtained. The Canon Non-Mydriatic Retinal Camera CR6-45NM was used to collect the fundus images. These photographs were graded at the Ocular Epidemiology Reading Center (OERC) at the University of Wisconsin-Madison for retinal microvascular characteristics, including central retinal arteriolar equivalent (CRAE), central retinal vein equivalent (CRVE), focal arteriolar narrowing (FAN), arterio-venous nicking (AVN), central arteriolar light reflex, and retinopathy signs (e.g., microaneurysms, retinal hemorrhages, cotton wool spots, and hard exudates and soft exudates). The dataset includes the generalized arteriolar narrowing which was measured through a quantified retinal artery and vein caliber/width using computer-based software. Other significant retinal conditions were also be noted, such as vascular occlusions in people with and without diabetes, and other eye conditions (e.g., cataracts).

**Table A2:** List of features selected for 5-year and 10-year stroke prediction models.

5-Year Stroke Prediction	10-Year Stroke Prediction
Age: Numeric (continuous) Gender: Boolean (0 = Female, 1 = Male) Race: Categorical (e.g., 1 = White, 2 = Black, 3 = Asian, 4 = Hispanic, etc.) BMI: Numeric (continuous) Smoking Status: Categorical (e.g., 0 = Never Smoked, 1 = Former Smoker, 2 = Current Smoker) Systolic Blood Pressure: Numeric (continuous) Diabetes Status: Boolean (0 = No, 1 = Yes) HDL Cholesterol: Numeric (continuous) LDL Cholesterol: Numeric (continuous) Total Cholesterol: Numeric (continuous) Hypertension: Boolean (0 = No, 1 = Yes) Hypertension Medication: Boolean (0 = No, 1 = Yes) History of Myocardial Infarction: Boolean (0 = No, 1 = Yes) History of Angina: Boolean (0 = No, 1 = Yes) History of Congestive Heart Failure: Boolean (0 = No, 1 = Yes) History of TIA: Boolean (0 = No, 1 = Yes) History of Any Heart Disease: Boolean (0 = No, 1 = Yes) The eye features selected are as follows: Cataract: Boolean (0 = No, 1 = Yes) Hemorrhages/Microaneurysm: Boolean (0 = No, 1 = Yes) AV Nicking: Boolean (0 = No, 1 = Yes) Hollenhorst Plaque: Boolean (0 = No, 1 = Yes) Central Retinal Artery Equivalent (CRAE): Numeric (continuous)	Age: Numeric (continuous) Gender: Boolean (0 = Female, 1 = Male) Race: Categorical (e.g., 1 = White, 2 = Black, 3 = Asian, 4 = Hispanic, etc.) Systolic Blood Pressure: Numeric (continuous) Microalbuminuria: Boolean (0 = No, 1 = Yes) 10-year CHD Risk: Coronary Artery Calcification: Numeric (continuous or ordinal based on Agatston score categories) Diabetes: Boolean (0 = No, 1 = Yes) Smoking Status: Categorical (e.g., 0 = Never Smoked, 1 = Former Smoker, 2 = Current Smoker) Family History of Heart Disease: Boolean (0 = No, 1 = Yes) Total Cholesterol: Numeric (continuous) HDL Cholesterol: Numeric (continuous) Cholesterol Medication: Boolean (0 = No, 1 = Yes) Hypertension Medication: Boolean (0 = No, 1 = Yes) Atrial Fibrillation: Boolean (0 = No, 1 = Yes) History of CHD, CHF, or PVD: Boolean (0 = No, 1 = Yes) ECG LVH (Left Ventricular Hypertrophy): Boolean (0 = No, 1 = Yes) The eye features selected are as follows: Cataract: Boolean (0 = No, 1 = Yes) Hemorrhages/Microaneurysm: Boolean (0 = No, 1 = Yes) AV Nicking: Boolean (0 = No, 1 = Yes) Hollenhorst Plaque: Boolean (0 = No, 1 = Yes) Central Retinal Artery Equivalent (CRAE): Numeric (continuous)

**Table A3:** Number of subjects in the training and testing dataset used for building 5-year stroke prediction.

	Total	Training Set	Test Set
Stroke Patients	171	121	50
Non-stroke Patients	5092	3673	1419

**Table A4:** Number of subjects in the training and testing dataset used for building 10-year stroke prediction.

	Total	Training Set	Test Set
Stroke Patients	242	187	55
Normal Patients	5499	3820	1679

**Table A5:** Risk score comparison with other popular risk scoring methods (10-year risk of stroke).

Risk Score	AUC
iHS Score	0.79 (0.78 to 0.80)
Framingham Score	0.73 (0.72 to 0.74)
CHADS2 Score	0.74 (0.73 to 0.75)
